# Spread of variants of epidemic disease based on the microscopic numerical simulations on networks

**DOI:** 10.1038/s41598-021-04520-0

**Published:** 2022-01-11

**Authors:** Yutaka Okabe, Akira Shudo

**Affiliations:** Department of Physics, Tokyo Metropolitan University, Hachioji, Tokyo 192-0397 Japan

**Keywords:** Biophysics, Mathematics and computing, Physics

## Abstract

Viruses constantly undergo mutations with genomic changes. The propagation of variants of viruses is an interesting problem. We perform numerical simulations of the microscopic epidemic model based on network theory for the spread of variants. Assume that a small number of individuals infected with the variant are added to widespread infection with the original virus. When a highly infectious variant that is more transmissible than the original lineage is added, the variant spreads quickly to the wide space. On the other hand, if the infectivity is about the same as that of the original virus, the infection will not spread. The rate of spread is not linear as a function of the infection strength but increases non-linearly. This cannot be explained by the compartmental model of epidemiology but can be understood in terms of the dynamic absorbing state known from the contact process.

## Introduction

Viruses of infectious diseases are constantly changing through mutation and become more diverse. It is expected that new variants of a virus will arise. Sometimes new variants appear and disappear. Other times, new variants may persist. It is important to study how viruses change and spread. Several novel variants of SARS-CoV-2, the virus that causes COVID-19, emerged in late 2020^1–3^. One of these, lineage B.1.1.7, was first detected in the UK in September 2020 and spread to multiple countries worldwide. This variant was labeled Alpha variant by the World Health Organization on 31 May 2021. The rapid spread of the UK variant (lineage B.1.1.7) suggests that it spreads more efficiently from person to person than the existing variants of SARS-CoV-2. The South Africa variant (B.1.351, Beta variant) and the Brazil variant (P.1, Gamma variant) were also identified in many countries. More recently, the India variant (B.1.617.2, Delta variant), which seems to be highly transmissible, is prevalent in some countries. A variant is of concern because it is more likely to spread, causing more severe disease, reducing the effectiveness of treatments or vaccines, or being more difficult to detect with current tests.

A variety of epidemiological models have been proposed to analyze the spread of the epidemic disease. Among them, a compartmental model is most commonly used, and each individual is supposed to be one of the possible types such as susceptible (S), infected (I), or recovered (R). The proportions of individuals in each type are taken to be continuous variables, and the rate equations among these proportions are then derived. The susceptible-infected-recovered (SIR) model, proposed in 1927 by Kermack and McKendrick^4^, is the most widely recognized basic model belonging to such a class. In “Differential equation of SIR model” section of Methods, we outline the differential equation of the SIR model^5–7^, because we perform simulations of the microscopic model of epidemic disease, which corresponds to the SIR model.

A given infected individual does not have an equal probability of infecting all others. Each individual only has contact with a small fraction of the total population, and the number of contacts that people have can vary greatly from one person to another. The connection between individuals can be described by a network. A network is a set of nodes (vertices) connected by edges. Two nodes share one edge, and from the point of view of one node, it has a direct relationship with the node connected by the edge, which is called a connected (neighboring) node. The number of edges of a node is referred to as the degree of that node.

Network science is the study of complex networks in the real world. It is based on mathematics and has applications in a wide range of fields, including statistical physics, computer science, electronics, ecology, economics, finance, and public health^8–10^. Here, we briefly describe what is relevant to the complex networks used in this study. The important development in network science was the work of Erdös and Rényi^11,12^ on graphs with random connections between nodes. This differs from the properties of the graphs actually appearing in the real world, but it is well-defined and a good testing ground for examining the properties of networks. Many real-world networks are neither uniform nor random. Small-worldness was pointed out; that is, a person can become a friend of a friend with only six intermediaries. Another point is the existence of a hub; there are nodes with a very large number of connections *k*, and the distribution of *k* is often scale-free, with a power distribution such as $k^{-a}$^13,14^. Barabási and Albert^14^ proposed an algorithm to create networks that exhibit scale-free properties. It is an algorithm that grows networks by selectively combining them. This mechanism is called “preferential attachment”. The emergence of the Barabási-Albert network has led to rapid progress in the study of complex networks across many fields.

The importance of the network structure in the analysis of epidemics was emphasized, and studies in several directions have been done^15–21^. In connection with COVID-19, stochastic simulations of the epidemic model have been performed^22,23^. The existence of absorbing states is one of the vital non-equilibrium processes on the complex network. Once the state has fallen into an absorbing state, the dynamics cannot escape from it^24–26^. The non-equilibrium absorbing phase transition is related to the directed percolation^27^. In epidemic spreading processes^6^, a fully healthy state can be regarded as an absorbing state in this sense. The contact process^28^ and the susceptible-infected-susceptible (SIS) model^29^ are often used to describe epidemic dynamics. It is well known that the SIS model can be mapped onto the logistic equation. In the epidemic scenario of the SIS model, individuals can be infected or susceptible. A phase transition between a disease-free (absorbing) phase and an active stationary phase is separated by an epidemic threshold. In the latter phase, a fraction of the population is infected. In the framework of the SIS model, the absence of an epidemic threshold was discussed for the spreading of infections on scale-free networks^15^.

The present authors^30^ performed simulations for the microscopic SIR model on networks, and in particular, the relationship between the SIR model for macroscopic quantities and the corresponding microscopic SIR model was discussed. For the network, we discussed the difference between random networks and scale-free networks, and the role of hubs in scale-free networks was elucidated. The simulation method follows the method of Herrmann and Schwartz^22^. They discussed the role of the absorbing state in the SIR model.

In this paper, we use a microscopic model of infectious disease transmission on networks to simulate the spread of variants. The simulation method is an extension of the method used in the absence of variants^30^. A related problem is that of “competing epidemics”^31–33^. When there are two competing diseases and the relative infectivity of the two diseases is different, a phase diagram of the behavior of infection was investigated^32^.

In the following, we describe the simulation method of microscopic SIR model including variants in “Method of simulation of the microscopic SIR model” section of Methods. As for networks, we treat two types of networks; the Erdös–Rényi (ER) network^11,12^, a random network, and the Barabási-Albert (BA) network^14^, a scale-free network. We will discuss the relationship between the infectious strength of the variant and the spread of the infection. We emphasize the relation to the non-equilibrium absorbing phase transition.

## Results

### Simulation of the microscopic model for the variants on the ER network

We first consider the case of the ER network, a random network. The total number of nodes (individuals) is $N=10{,}000$ and the average number of degrees is $\langle k \rangle =8$. To perform the simulation of the microscopic model for the spread of infection, we choose the probability *p* of infection as $p=9/200$, which leads to $\beta = \langle k \rangle p = 0.36$ in terms of a rate constant of the SIR model, which will be explained in “Differential equation of SIR model” section of Methods. The average infected period, $1/\gamma$, is chosen as 5.0 days, which is realized by a Poisson distribution with an average value of 5.0. The basic reproduction number $R_0=\beta /\gamma$, Equation (7), becomes $R_0=1.8$.

**Figure 1:**
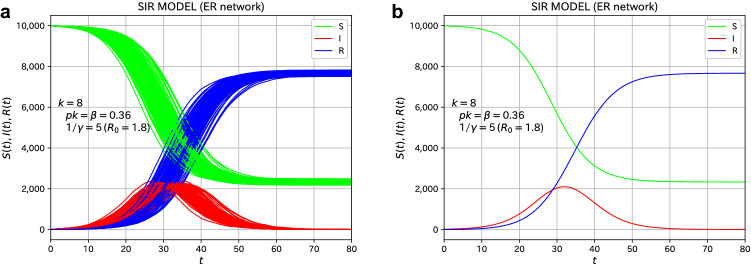
The simulational results of the microscopic SIR model on the ER network (reference system with no variants). (**a**) the plot of all 100 samples assumed to be infected with the virus with $R_0=1.8$. (**b**) the average over 100 samples. Initially, 10 individuals were set to be infected.

As a reference system, we deal with the case where there are no variants. The initial condition ($t=0$) is that 10 randomly selected individuals are infected. The time evolution of the number of individuals of the three types (S, I, and R) is shown in Fig. 1. We performed simulations for 100 samples, and the time evolution of all samples are plotted in Fig. 1a. We observe a variation in the time evolution for each sample. On the other hand, Fig. 1b is an average plot of 100 samples. The number of infected individuals (I) increases with time, reaches a peak, and gradually decreases, which is the same behavior as that for the SIR model of the differential equation. The microscopic SIR model on a random network is considered to reproduce the macroscopic SIR model almost quantitatively. The final value of the total number of infected individuals ($R(\infty )$) is 0.732 from Eq. (8), the final size equation of the SIR model^34^, in the case of $R_0=\beta /\gamma =1.8$, and the measured value for the present microscopic model on the ER network is about 0.77. As a related argument, the final size was discussed in the framework of a stochastic epidemic model^35^. In the Reed–Frost model^36,37^, which is a typical example of a stochastic epidemic model, the final size can be treated analytically. Britton et al. discussed the final size of stochastic epidemic models on networks^38^. In the previous study^30^, for a small number of initially infected individuals, some examples showed the behavior such that the infection vanishes quickly and does not spread throughout the network. This behavior is regarded as the absorbing state^24–26^ in the contact process^28^. We chose 10 initially infected cases to avoid the situation of the absorbing state.

**Figure 2:**
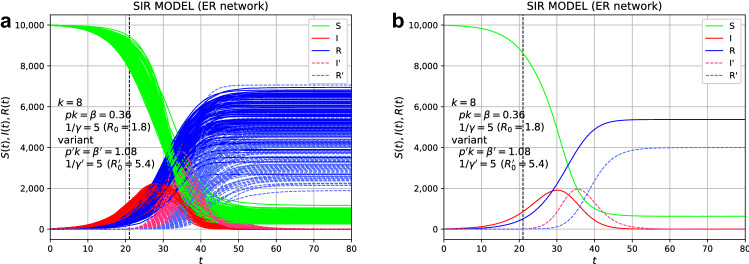
Effects of variants for the spread of the epidemic disease on the ER network. Suppose that 10 individuals are infected with the variant ($R_0'=5.4$) by an external factor at $t=21$. (**a**) The plot of all 100 samples. (**b**) The average of the 100 samples. The red and blue dashed lines indicate the values of the variant.

We next consider the effects of variants. Suppose that 10 susceptible individuals are infected with the variant due to external factors at $t=21$. We choose 10 individuals randomly among the non-infected. The variant is assumed to be 3.0 times more infectious with $\beta '=1.08$, and the average infection period is chosen as the same value as that of the original virus, $1/\gamma '=5.0$. Then, the basic reproduction number of the variant becomes $R_0'=5.4$. The situation at $t=21$ is that about 900 individuals are infected, 500 individuals are recovered, and 8600 individuals are not infected. In Fig. 2, the number of individuals infected with the variant (I’) and those who recovered from the variant (R’) are shown in the dashed line. The time when the variant is added is indicated by the vertical black dashed line. The variant infection starts to spread at $t=21$, but as can be seen in Fig. 2a, which plots all 100 samples, there is a large sample dependence. Figure 2b, which is the average of 100 samples, shows the general trend. The value of $R(\infty )$ represents the final total number of infected individuals of the original virus, and the value of $R'(\infty )$ represents the total number of individuals infected with the variant. The value of $R(\infty )$ slightly decreases compared to the case without the variant. Compare the solid blue line in Fig. 2b with that in Fig. 1b.

**Figure 3:**
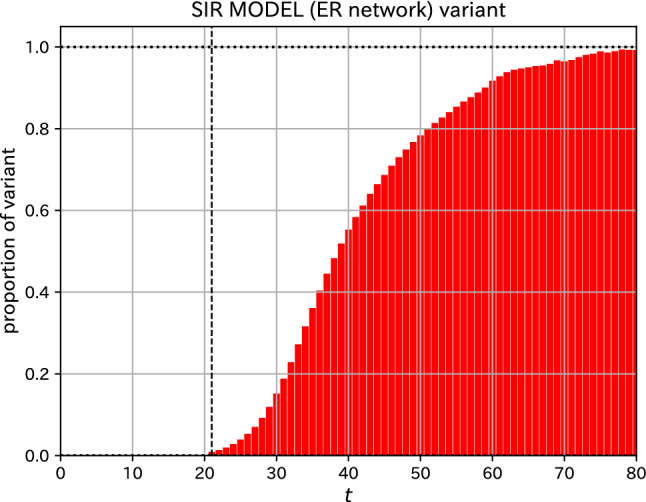
Time variation of the proportion of variants among the infected. The data shown in Fig. 2 was used to make this graph.

An increase and decrease in the proportion of variants are often discussed. The calculated time variation of the proportion of variants from the data in Fig. 2 is plotted in Fig. 3. An increase in the proportion of variants is observed, which means that the infection of variants has spread.

**Figure 4:**
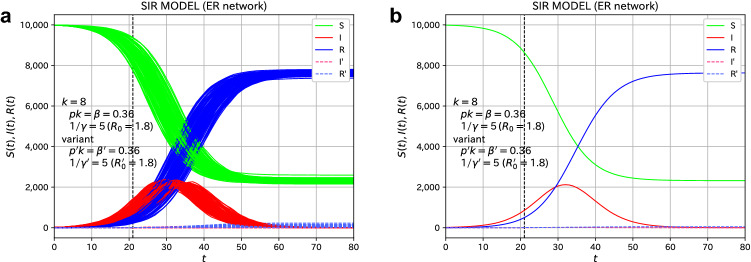
Effects of variants for the spread of the epidemic disease on the ER network. Suppose that 10 individuals are infected with the original virus ($R_0'=1.8$) by an external factor at $t=21$. (**a**) The plot of all 100 samples. (**b**) The average of the 100 samples. The red and blue dashed lines are additional values.

For comparison, let us consider the case where the infectivity of the variant to be added is the same as that of the original species. Suppose that at $t=21$, 10 people are infected by the variant with $R_0'=1.8$, the same as the original species. Figure 4 shows the time evolution of the infected individuals. As can be seen from Fig. 4, in this case, the variant infection does not spread for all 100 samples. The spread of the additional variant is regarded as in the absorbing state. The addition of 10 infected individuals with the variant to 900 infected individuals will have little effect.

We have shown that when the infectivity of the variant is strong, even if a small number of individuals are infected, it leads to the spread of the infection. On the other hand, when the infectivity of the variant is comparable to that of an original virus, it does not spread, and this is a dynamic effect. So what happens when the infectivity is intermediate? The results of measurements for intermediate values of the basic reproduction number of variants are shown in the supplementary information. There, the time evolution graphs for the variants with $R_0'=4.95$, $R_0'=4.5$, $R_0'=4.05$, $R_0'=3.6$, $R_0'=3.15$, $R_0'=2.7$, and $R_0'=2.25$ are also given.

**Figure 5:**
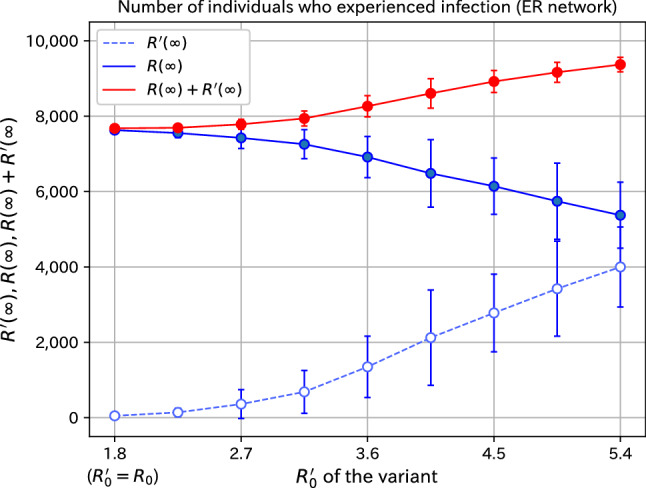
Dependence of the final number of infected individuals on $R_0'$ of the variant. The infectivity of the variant is the same as that of the original virus for $R_0'=1.8$.

We examine the behavior of the spread of variants systematically. The dependence of the final number of infected on the basic reproduction number $R_0'$ of the variant is plotted in Fig. 5. The number of infected individuals of the variant is shown by the dashed blue line and the number of infected individuals of the original species by the solid blue line. The standard deviation of the 100 samples is indicated by the error bars. The infectivity of the variant is the same as that of the original virus for $R_0'=1.8$. It can be seen that the number of infected individuals of the variant increases with the infectivity and the fluctuation also increases. The increase is not linear; that is, when the infectivity becomes slightly larger, the spread of infection is small, but as the infectivity further increases, the increase is non-linear. On the other hand, the number of the infected individuals with the original species decreases slightly. This is because individuals who would have been infected with the original species if there had been no new infections of the variant will be infected with the variant. The sum of the infected individuals with the variant and those with the original species is shown by the red line, and the sum increases. However, the fluctuation of the sum is not necessarily large.

To summarize what we have shown in this subsection, we can say the following. When the number of infected individuals reaches about 900 (the total number is 10,000), a small number of individuals infected with the variant are added. If the variant is highly infectious, it will spread to the wide space from the 10 additional infected individuals. By systematically examining the variants whose basic reproduction numbers are 1.0, 1.25, 1.5, 1.75, 2.0, 2.25, 2.5, 2.75, and 3.0 times that of the original species, we showed that the infection increases non-linearly with the basic reproduction number. The lack of infection to spread in the case of variants whose infectivity is not much different from that of the original species is due to the dynamic effect of infection, that is, the absorbing state.

### Simulation of the microscopic model for the variants on the BA network

Next, we consider the case of the BA network, a scale-free network. We choose the network which is similar to the case of the ER network. The total number of nodes (individuals) is $N=10{,}000$ and the average number of degrees is $\langle k \rangle =8$. The spread of infection is more rapid in the case of scale-free networks because of a hub structure^30^. Thus, for the probability of infection *p*, we choose a value smaller than that for the ER network, that is, $p=1/25$. This leads to $\beta = \langle k \rangle p = 0.32$ in terms of a rate constant of the SIR model. The average infected period, $1/\gamma$, is again chosen as 5.0 days. Then, the basic reproduction number becomes $R_0=\beta /\gamma =1.6$.

**Figure 6:**
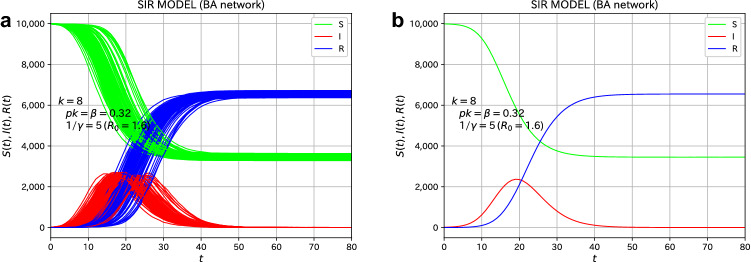
The simulational results of the microscopic SIR model on the BA network (reference system with no variants). (**a**) The plot of all 100 samples assumed to be infected with the virus with $R_0=1.6$. (**b**) The average over 100 samples. Initially, 10 individuals were set to be infected.

We treat the case where there are no variants as a reference system. The conditions are the same as the case of the ER network. The time evolution of the number of individuals of the three types (S, I, and R) is shown in Fig. 6. We performed simulations for 100 samples. We plot the time evolution of all 100 samples in Fig. 6a, whereas the average of 100 samples is plotted in Fig. 6b.

**Figure 7:**
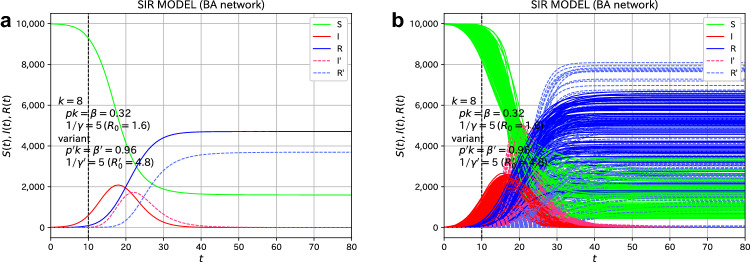
Effects of variants for the spread of the epidemic disease on the BA network. Suppose that 10 individuals are infected with the variant ($R_0'=4.8$) by an external factor at $t=10$. (**a**) The plot of all 100 samples. (**b**) The average of the 100 samples. The red and blue dashed lines indicate the values of variant.

We turn to the investigation of the effects of variants. Suppose that 10 susceptible individuals are infected with the variant due to external factors at $t=10$. The variant is assumed to be 3.0 times more infectious with $\beta '=0.96$. The average infection period is chosen as $1/\gamma '=5.0$, which leads to $R_0'=4.8$. The situation at $t=10$ is that about 620 individuals are infected, 100 individuals are recovered, and 9280 individuals are not infected. This is similar to the case of ER network at $t=21$ shown in Fig. 2. We plot the time variation of the spread of infection on the BA network in Fig. 7. The number of individuals infected with the variant (I’) and those who recovered from the variant (R’) are shown in the dashed line. The overall behavior of the BA network is similar to the case of the ER network.

**Figure 8:**
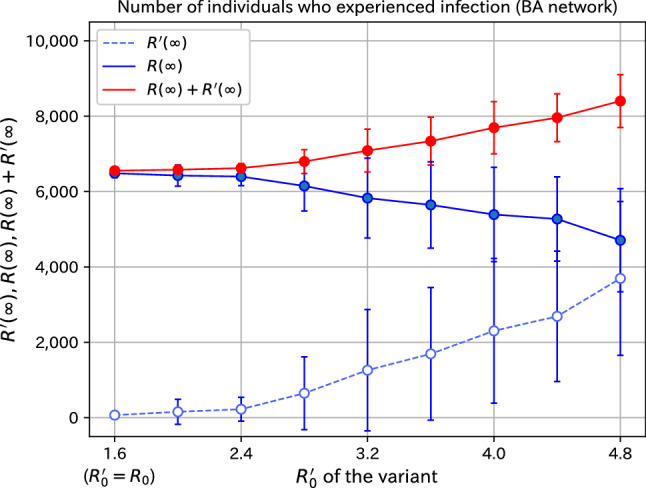
Dependence of the final number of infected individuals on $R_0'$ of the variant. The infectivity of the variant is the same as that of the original virus for $R_0'=1.6$.

The results of measurements for various $R_0'$ of the variant on the BA network are shown in the supplementary information. There, the time evolution graphs for the variants with $R_0'=4.4$, 4.0, 3.6, 3.2, 2.8, 2.4, 2.0, and 1.6 are also given. The basic reproduction number $R_0'=1.6$ is the same number as the original virus. Summarizing the results for various $R_0'$, we plot the dependence of the final number of infected on the basic reproduction number $R_0'$ of the variant in Fig. 8. The number of infected individuals of the variant is shown by the dashed blue line, the number of infected individuals of the original species by the solid blue line, and the sum of the two by the red line. The standard deviation of the 100 samples is indicated by the error bars. The infectivity of the variant is the same as that of the original virus for $R_0'=1.6$. From Fig. 8, we observe that if the variant is highly infectious, the number of individuals infected with the variant increases. From the systematical study with infectivity of 1.0, 1.25, 1.5, 1.75, 2.0, 2.25, 2.5, 2.75, and 3.0 times, we see that the effect is not simply proportional to the infectivity. We also observe that the number of individuals infected with the original species decreases slightly and the total number of the infected increases. The fluctuation in the number of infected individuals becomes significant when the infectivity of the variant is strong. However, the fluctuation of the sum of the variant and the original species is not necessarily significant. The effect of a variant on the BA network is essentially the same as that of a variant on the ER network. The characteristics of the scale-free network are not particularly evident.

## Discussion

We studied the spread of variants of the virus by performing numerical simulations of the microscopic model on the network. In the middle of a simulation of infectious disease on the network, we added a variant that is more transmissible than the original lineage. When a highly infectious variant is added, the variant spreads quickly. It is noteworthy that the rate of spread is not linear in the infectivity of the variant but it is non-linear. If a variant is slightly more transmissible than the original lineage, the virus does not spread widely. It is related to a non-equilibrium phase transition of the epidemic dynamics on the complex network between a disease-free (absorbing) state and an active stationary phase^24–26^. This cannot be accounted for by the compartmental model of epidemiology. We performed simulations both on the ER model, a random network, and the BA model, a scale-free network. It was shown that the existence of the hub for a scale-free network stimulates the rapid increase of the infection^30^. But the effect of the variants for the BA network is essentially similar to the case of the ER network. It should also be noted that there is a lot of fluctuation in the spread of the more infectious variants. The greater fluctuation is due to the network structure. In summary, the reason why the more infectious variants spread, while the less infectious variants do not, has been clarified.

Although the present work is for a model system with some parameters, we may learn some lessons for a real-world strategy to fight against the pandemic. The spread of a slightly more infectious variant is not significant. Variants that are more infectious than the original lineage will spread the infection in a non-linear fashion. Of course, whether or not the variant is severe is an important factor. It can be said that the major role of overseas quarantine is to identify variants. For highly infectious variants, it is necessary to identify them by genetic testing and to isolate infected individuals. To conduct special contact tracing is of particular importance in the case that they spread throughout the city. Those variants must be monitored more carefully. Measures need to be taken to control the spread of the virus and its variants. Travel restrictions can be effective in reducing the spread of the virus. Contact with people over long distances is associated with the presence of hubs in the network. As shown in the previous paper^30^, isolation of hub sites can lead to transmission control. This is also the case for variants, for which travel restrictions are effective. However, to ultimately control the epidemic, it may be necessary to greatly accelerate vaccine roll-out.

## Methods

### Differential equation of SIR model

We perform simulation of the microscopic model of epidemic disease, which corresponds to the compartmental model of macroscopic variables. We consider SIR model^4–7,30^ as a compartmental model.

We consider a closed society of *N* individuals and classify individuals into three types: susceptible (S), infected (I), and recovered (R). Infected individuals can only transmit the virus to susceptible individuals. Once infected individuals have recovered (or have passed away), they can no longer infect others and cannot be reinfected. Let *S*(*t*), *I*(*t*), and *R*(*t*) denote the number of individuals in three states S, I, and R as a function of time *t*. In the SIR model, the time evolution is described by the following simultaneous differential equations.

1 $$\begin{aligned} \frac{dS(t)}{dt}= & {} - \frac{\beta }{N} S(t)I(t), \end{aligned}$$

2 $$\begin{aligned} \frac{dI(t)}{dt}= & {} \frac{\beta }{N} S(t)I(t)- \gamma I(t), \end{aligned}$$

3 $$\begin{aligned} \frac{dR(t)}{dt}= & {} \gamma I(t), \end{aligned}$$

where $\beta$ is the rate of infection and $\gamma$ the rate of recovery (the rate of quarantine). We consider that each person is in contact with *k* persons per unit time (day), and the probability of infection for each contact is set as *p*. Then, $\beta$ is given as *kp*. We assume that the total number of individuals is set to be constant such that

4 $$\begin{aligned} S(t)+I(t)+R(t)=N. \end{aligned}$$

Here, we do not consider the birth and death processes.

The SIR Eqs. (1)$\sim$(3), is said to have been given an exact solution by Harko *et al.* in 2014^39^. However, from the standpoint of differential equation theory, it was known to be solvable before that^40^. From Eqs. (1) and (2), we obtain

5 $$\begin{aligned} \frac{dI}{dS} = \frac{- (\beta /N) S + \gamma }{(\beta /N) S} = -1 + \frac{\gamma }{\beta } \Big ( \frac{N}{S} \Big ). \end{aligned}$$

The integration with respect to *S* yields

6 $$\begin{aligned} I = - S + \frac{\gamma }{\beta } N \ln S + C, \end{aligned}$$

where *C* is an integral constant. If we insert Eq. (6) into Eq. (1), we obtain a closed expression for *S*(*t*). We note that the ratio of $\beta$ and $\gamma$ appears for rate constants. The number $R_0$ defined by

7 $$\begin{aligned} R_0 = \frac{\beta }{\gamma } \end{aligned}$$

is known as the basic reproduction number^6,41,42^, and the number of infected individuals increases when $R_0 > 1$, whereas it decreases when $R_0 < 1$.

It is noteworthy that *I*(*t*) is the number of infected individuals, not the number of new infected individuals announced every day. It should be noted that the number of new infections can be discussed in the framework of the SIR model^7^.

We here make a comment on the final size equation. We have the relation for the final value of $R(\infty )$. In the limit of $N_1 \rightarrow N$, we obtain

8 $$\begin{aligned} 1 - R(\infty )/N = \exp \Big [- \frac{\beta }{\gamma }(R(\infty )/N) \Big ]. \end{aligned}$$

This relation is known as the final size equation^34^.

### Method of simulation of the microscopic SIR model

Let us consider a simulation of microscopic infectious disease propagation in a network, in which each node assumes the state of infectious disease and changes its state stochastically. The rules for updating the state correspond to the macroscopic SIR model given by the differential equations, and the parameters are chosen to be equivalent so that the macroscopic and microscopic SIR models can be compared. The actual procedure, which is similar to that by Herrmann and Schwartz^22^, is as follows^30^:

1. Generate a network.
2. At $t=0$, an individual or individuals are initially infected (I).
3. A susceptible individual (S) will be infected (I) with a probability *p* if a connecting individual (one of *k*) is infected (I). We do this for all nodes (non-infected) and for all connections (*k*) of each node. In terms of the SIR model, the parameter $\beta$ is $\beta =kp$.
4. An infected individual (I) will recover (R) in $1/\gamma$ days on average. The infected period is chosen by a Poisson distribution with an average of $1/\gamma$.
5. At each time *t*, the processes 3, 4 are repeated.
6. The time sequence obtained from the above procedure is regarded as a single sample. Simulations are performed for several samples.

In the simulation to study the effect of the variant, we will assume that some individuals are infected by the variant at time $t=t_{\mathrm{var}}$. We will denote by I′ those who are infected by the variant and by R′ those who recover from the variant. Modify the processes 2–4 of the simulation as follows:

2′ At $t=t_{\mathrm{var}}$, an individual or individuals are infected with the variant (I′).
3′ A susceptible individual (S) will be infected by the original virus (I) with a probability *p* if a connecting individual is infected by the original virus (I). If a connecting individual is infected by the variant (I′), a susceptible individual (S) will be infected by the variant (I′) with a probability $p'.$
4′ An infected individual by the original virus (I) will be recovered (R) in $1/\gamma$ days on average. The infected period is chosen by a Poisson distribution with an average of $1/\gamma$. An infected individual by the variant of the virus (I′) will be recovered (R′) in $1/\gamma '$ days on average.

Then, the condition in Eq. (4) that the total number of individuals of various types is conserved is modified as

9 $$\begin{aligned} S(t)+I(t)+R(t)+I'(t)+R'(t)=N. \end{aligned}$$

We perform the simulation of the microscopic model using the procedures described above. We here make a short comment on the distribution of the infection period. The geometric distribution for a discrete-time model was used to evaluate the impact of waiting-time distributions on epidemiological processes^43^. On the other hand, Herrmann and Schwartz^22^ used a distribution referring to the covid-19 data to perform microscopic simulations on complex networks. In the present paper, we employed the Poisson distribution as a model, which does not have a very long time tail. It is the effect of the variants that we are discussing.

## Supplementary Information

Supplementary Information.

## References

[1] Davies NG, Abbott S, Barnard RC (2021). Estimated transmissibility and impact of SARS-CoV-2 lineage B.1.1.7 in England. Science.

[2] Davies, N. G., Jarvis, C. I., CMMID COVID-19 Working Group, Edmunds, W. J., Jewell, N. P., Diaz-Ordaz, K. & Keogh, R. H. Increased mortality in community-tested cases of SARS-CoV-2 lineage B.1.1.7. *Nature* **593**, 270-274 (2021). 10.1038/s41586-021-03426-1 PMC9170116 33723411

[3] Volz E, Mishra S, Chand M (2021). Assessing transmissibility of SARS-CoV-2 lineage B.1.1.7 in England. Nature.

[4] Kermack WO, McKendrick AG (1927). A contribution to the mathematical theory of epidemics, I. Proc. R. Soc. Lond. A.

[5] Bailey NTJ (1975). The Mathematical Theory of Infectious Diseases and Its Applications.

[6] Diekmann O, Heesterbeek JAP (2000). Mathematical Epidemiology of Infectious Diseases: Model Building, Analysis and Interpretation.

[7] Okabe Y, Shudo A (2020). A mathematical model of epidemics—a tutorial for students. Mathematics.

[8] Caldarelli G, Catanzaro M (2012). Networks: A Very Short Introduction.

[9] Barabási A-L (2016). Network Science.

[10] Newman M (2018). Networks.

[11] Erdös P, Rényi A (1959). On random graphs I. Publ. Math..

[12] Erdös P, Rényi A (1960). On the evolution of random graphs. Publ. Math. Inst. Hungar. Acad. Sci..

[13] De Solla Price DJ (1965). Networks of scientific papers. Science.

[14] Barabási A-L, Albert R (1999). Emergence of scaling in random networks. Science.

[15] Pastor-Satorras R, Vespignani A (2001). Epidemic spreading in scale-free networks. Phys. Rev. Lett..

[16] Dezsö Z, Barabási A-L (2002). Halting viruses in scale-free networks. Phys. Rev. E.

[17] Newman MEJ (2002). Spread of epidemic disease on network. Phys. Rev. E.

[18] Hufnagel L, Brockmann D, Geisel T (2004). Forecast and control of epidemics in a globalized world. PNAS.

[19] Keeling MJ, Eames KTD (2005). Networks and epidemic models. J. R. Soc. Interface.

[20] Castellano C, Pastor-Satorras R (2012). Competing activation mechanisms in epidemics on networks. Sci. Rep..

[21] Pastor-Satorras R, Castellano C. Van, Mieghem P, Vespignani A (2015). Epidemic processes in complex networks Rev. Mod. Phys..

[22] Herrmann HA, Schwartz J-M (2020). Why COVID-19 models should incorporate the network of social interactions. Phys. Biol..

[23] Choi, K., Choi, H. & Kahng, B. Covid-19 epidemic under the K-quarantine model: Network approach. arXiv:2010.07157 (2020). 10.1016/j.chaos.2022.111904 PMC8831130 35169382

[24] Marro J, Dickman R (1999). Nonequilibrium Phase Transitions in Lattice Models (Collection Alea-Saclay: Monographs and Texts in Statistical Physics).

[25] Henkel M, Hinrichsen H, Lubeck S (2008). Non-Equilibrium Phase Transitions: Volume 1: Absorbing Phase Transitions.

[26] Mata AS (2021). An overview of epidemic models with phase transitions to absorbing states running on top of complex networks. Chaos.

[27] Broadbent SR, Hammersley JM (1957). Percolation processes: I. Crystals and mazes. Proc. Camb. Philos. Soc..

[28] Harris TE (1974). Contact interactions on a lattice. Ann. Probab..

[29] Anderson, R. M. & May, R. M. *Infectious Diseases in Humans*, (Oxford University Press, Oxford, 1992). Contact Interactions on a Lattice. *Ann. Probab.* **2**, 969–988 (1974).

[30] Okabe Y, Shudo A (2021). Microscopic numerical simulations of epidemic models on networks. Mathematics.

[31] Newman MEJ (2005). Threshold effects for two pathogens spreading on a network. Phys. Rev. Lett..

[32] Karrer D, Newman MEJ (2011). Competing epidemics on complex networks. Phys. Rev. E.

[33] Sahneh FD, Scoglio C (2014). Competitive epidemic spreading over arbitrary multilayer networks. Phys. Rev. E.

[34] Metz JAJ, Diekmann O (1986). The Dynamics of Physiologically Structured Populations.

[35] Britton T (2010). Stochastic epidemic models: A survey. Math. Biosci..

[36] Schwabe CW, Riemann HP, Franti CE (1977). Epidemiology in Veterinary Practice.

[37] Abbey H (1952). An examination of the Reed–Frost theory of epidemics. Hum Biol..

[38] Britton T, Janson S, Martin-Löf A (2007). Graphs with specified degree distributions, simple epidemics and local vacination strategies. Adv. Appl. Prob..

[39] Harko T, Lobo FSN, Mak MK (2014). Exact analytical solutions of the susceptible-infected-recovered (SIR) epidemic model and of the SIR model with equal death and birth rates. Appl. Math. Comput..

[40] Hirsch MW, Smale S, Devaney RL (2010). Differential Equations, Dynamical Systems, and an Introduction to Chaos.

[41] Diekmann O, Heesterbeak JAP, Metz JAJ (1990). On the definition and the computation of the basic reproduction ratio R0 in models for infectious diseases in heterogeneous populations. J. Math. Biol..

[42] Dietz K (1993). The estimation of the basic reproduction number for infectious diseases. Stat. Methods Med. Res..

[43] Hernandez-Ceron N, Feng Z, Castillo-Chavez C (2013). Discrete epidemic models with arbitrary stage distributions and applications to disease control. Bull. Math. Biol..

