# Supplementary material for: Spread of variants of epidemic disease based on the microscopic numerical simulations on networks
Source: Sci Rep. 2022 Jan 11;12:523. doi: 10.1038/s41598-021-04520-0 (PMC8752609; doi:10.1038/s41598-021-04520-0)
Supplement: Supplementary file 1 — Supplementary Information. [file 41598_2021_4520_MOESM1_ESM.pdf]

Spread of variants of epidemic disease based on the microscopic numerical simulations on networks: Supplementary Information

Yutaka Okabe* and Akira Shudo+

Department of Physics, Tokyo Metropolitan University, Hachioji, Tokyo 192-0397, Japan
*okabe@phys.se.tmu.ac.jp
+shudo@tmu.ac.jp

In the supplementary information, we show graphs of simulations on the complex networks for the spread of variants of viruses for various parameters. We perform the simulation of the microscopic SIR model[1,2]. We consider both the Erdös-Rényi (ER) network[3,4], a random network, and the Barabási-Albert (BA) network[5]. A detailed analysis is given in the main text.

Simulation of the microscopic model for the variants on the ER network

We first consider the case of the ER network, a random network. The total number of nodes (individuals) is $N = 10000$ and the average number of degrees is $\langle k \rangle = 8$. To perform the simulation of the microscopic model for the spread of infection, we choose the probability p of infection as $p = 9/200$, which leads to $\beta = \langle k \rangle p = 0.36$ in terms of a rate constant of the SIR model. The average infected period, $1/\gamma$, is chosen as 5.0 days, which is realized by a Poisson distribution with the average value of 5.0. The basic reproduction number becomes $R_0 = \beta/\gamma = 1.8$.

As a reference system, we deal with the case where there are no variants. The initial condition ($t = 0$) is that 10 randomly selected individuals are infected. The time evolution of the number of individuals of the three types (S, I, and R) is shown in Figure S1. We performed simulations for 100 samples, and the time evolution of all samples are plotted in Figure S1(a). We observe a variation in the time evolution for each sample. On the other hand, Figure S1(b) is an average plot of 100 samples. The number of infected individuals (I) increases with time, reaches a peak, and gradually decreases, which is the same behavior as that for the SIR model of the differential equation. In the case of a small number of initially infected individuals, some example shows the behavior such that the infection vanishes quickly and does not spread throughout the network. This behavior is regarded as the absorbing state[6,7] in the contact process[8]. We chose 10 initially infected cases to avoid the situation of the absorbing state.

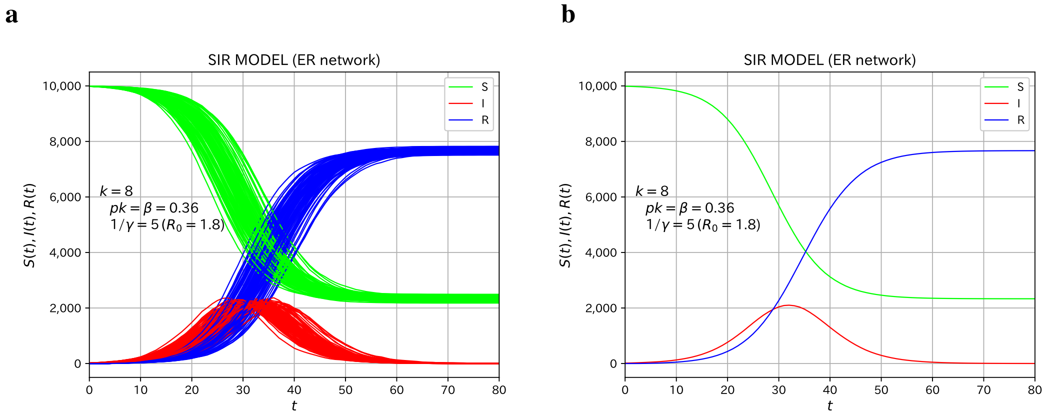

Figure S1. The simulational results of the microscopic SIR model on the ER network (reference system with no variants). (a) the plot of all 100 samples assumed to be infected with the virus with $R_0 = 1.8$. (b) the average over 100 samples. Initially, 10 individuals were set to be infected.

Next, consider the effects of variants. Suppose that 10 susceptible individuals are infected with the variant due to external factors at $t = 21$. We choose them randomly among the non-infected. The variant is assumed to be 3.0 times more infectious with $\beta' = 1.08$, and the average infection period is chosen as the same value as that of the original virus, $1/\gamma' = 5.0$. Then, the basic reproduction number of the variant becomes $R'_0 = 5.4$. The situation at $t = 21$ is that about 900 people are infected, 500 people are recovered, and 8600 people are not infected. In Figure S2, the number of individuals infected with the variant (I') and those who recovered from the variant (R') are shown in the dashed line. The time when the variant is added is indicated by the vertical black dashed line. The variant infection starts to spread at $t = 21$, but as can be seen in Figure S2(a), which plots all 100 samples, there is a large sample dependence. Figure S2(b), which is the average of 100 samples, shows the general trend. The value of $R(\infty)$ represents the final total number of infected individuals of the original virus, and the value of $R'(\infty)$

represents the total number of individuals infected with the variant. The value of $R(\infty)$ slightly decreases compared to the case without the variant. Compare the solid blue line in Figure S2(b) with that in Figure S1(b).

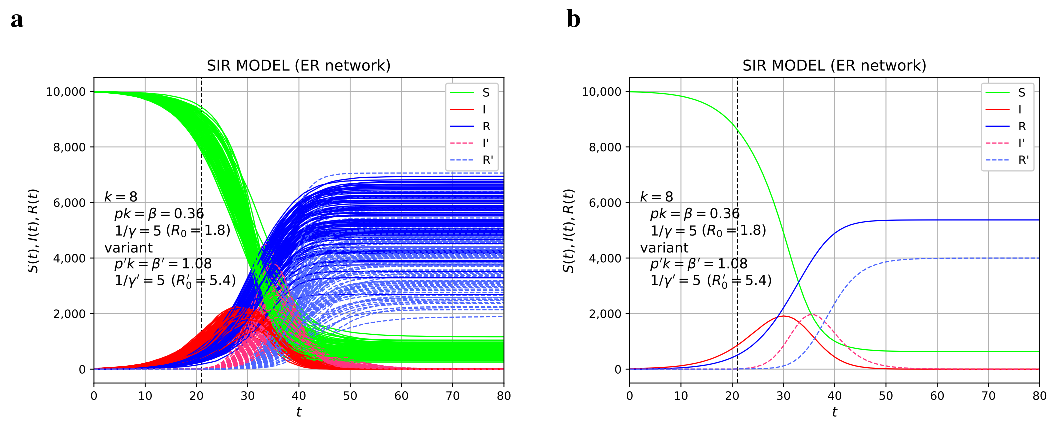

Figure S2. Effects of variants for the spread of the epidemic disease on the ER network. Suppose that 10 individuals are infected with the variant ($R'_0 = 5.4$) by an external factor at $t = 21$. (a) the plot of all 100 samples. (b) the average of the 100 samples. The red and blue dashed lines indicate the values of the variant.

In Figure S3, we show the raw data of the spread of the epidemic disease with the variants with various strengths of infection. All the conditions are the same as in Figure S2 except for the value of β', that is R'_0, of the variant. We plot the data of $R'_0 = 4.95$, 4.5, 4.05, 3.6, 3.15, 2.7, 2.25, and 1.8 in (a1,b1), (a2,b2), (a3,b3), (a4,b4), (a5,b5), (a6,b6), (a7,b7), and (a8,b8), respectively. They are 2.75, 2.5, 2.25, 2.0, 1.75, 1.5, 1.25, and 1.0 times that of the value of the original virus. As the infectivity of the variant becomes weaker, the spread of the infection gradually becomes smaller.

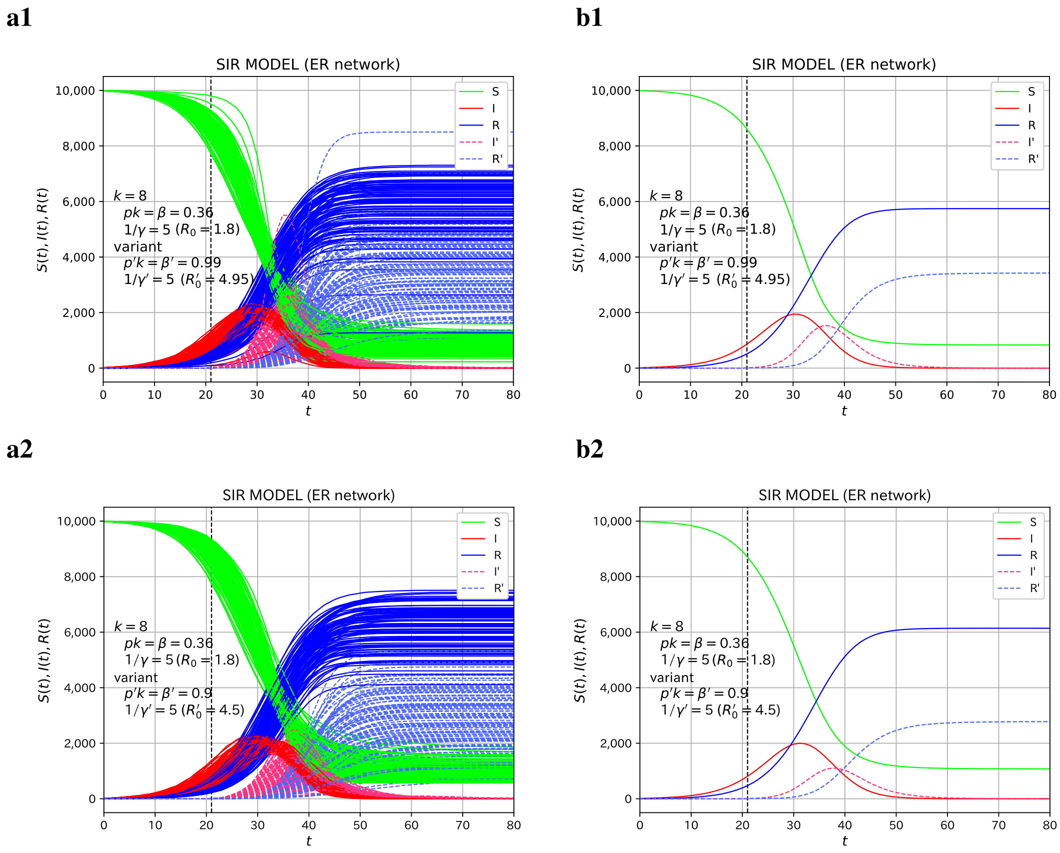

a3

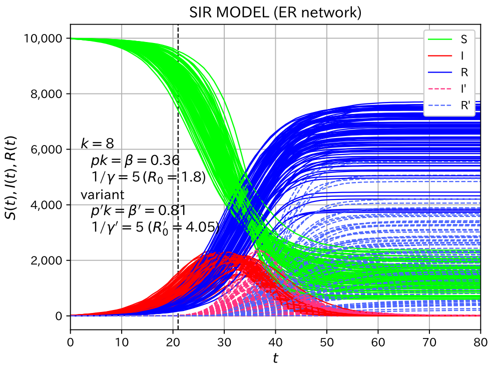

b3

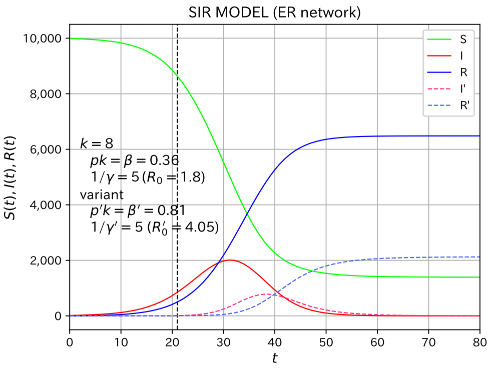

a4

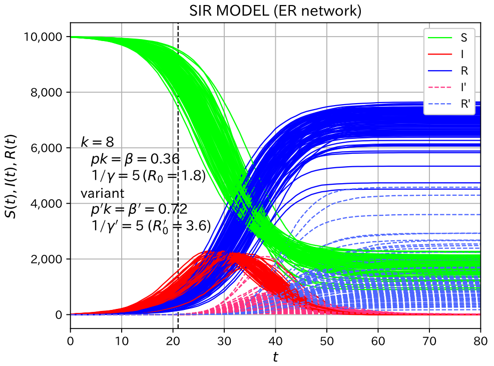

b4

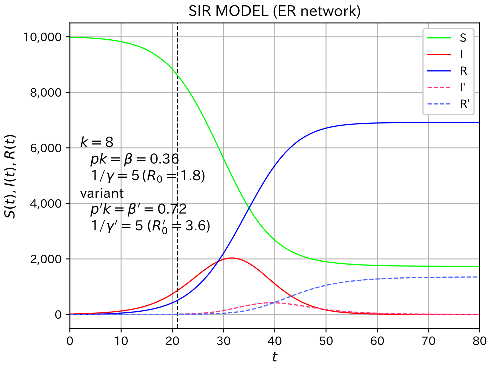

a5

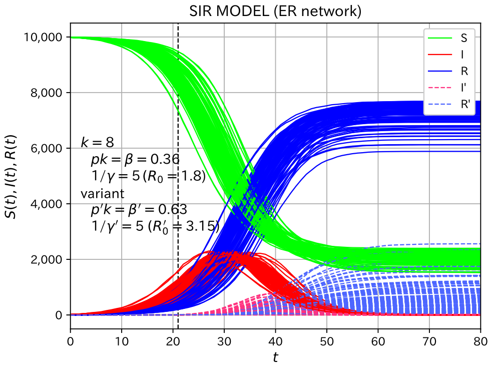

b5

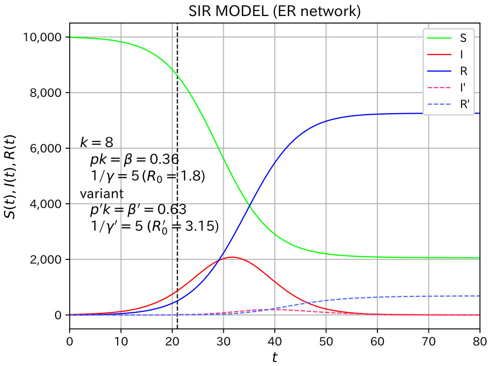

a6

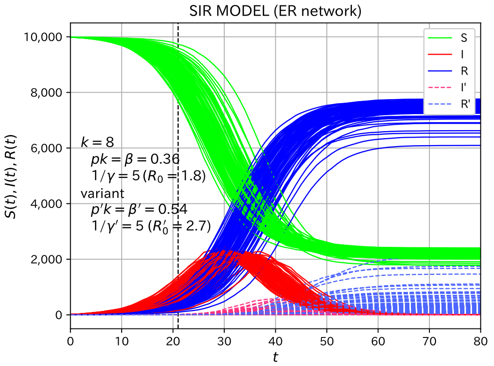

b6

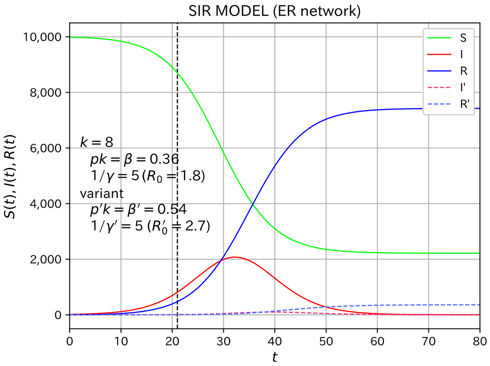

a7

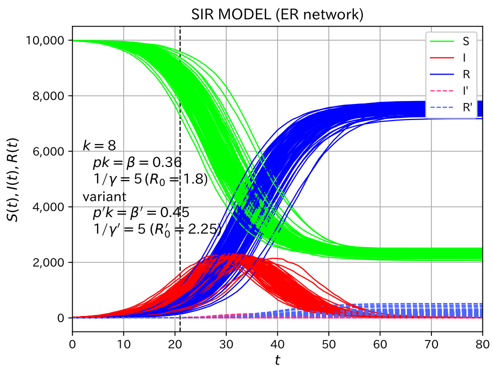

b7

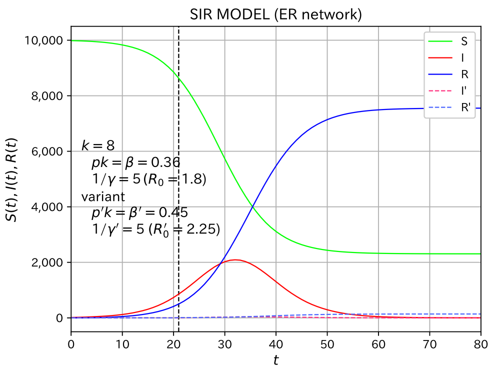

a8

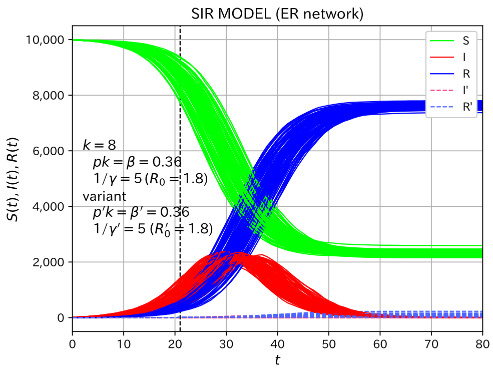

b8

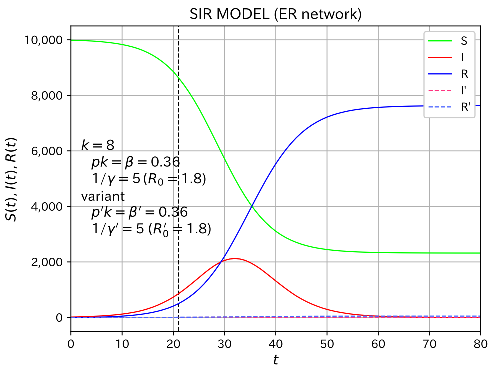

Figure S3. Effects of variants for the spread of the epidemic disease on the ER network. Suppose that 10 individuals are infected with the variant by an external factor at $t = 21$. (a1,b1) the plot of all 100 samples and the average of the 100 samples for $R_0' = 4.95$. (a2,b2) those for $R_0' = 4.5$. (a3,b3) those for $R_0' = 4.05$. (a4,b4) those for $R_0' = 3.6$. (a5,b5) those for $R_0' = 3.15$. (a6,b6) those for $R_0' = 2.7$. (a7,b7) those for $R_0' = 2.25$. (a8,b8) those for $R_0' = 1.8$. The red and blue dashed lines indicate the values of the variant.

Simulation of the microscopic model for the variants on the BA network

Next, we consider the case of the BA network, a scale-free network. We choose the network which is similar to the case of the ER network. The total number of nodes (individuals) is $N = 10000$ and the average number of degrees is $\langle k \rangle = 8$. The spread of infection is more rapid in the case of scale-free networks. Thus, for the probability of infection p, we choose a value smaller than that for the ER network, that is, $p = 1/25$. This leads to $\beta = \langle k \rangle p = 0.32$ in terms of a rate constant of the SIR model. The average infected period, $1/\gamma$, is again chosen as 5.0 days. Then, the basic reproduction number becomes $R_0 = \beta/\gamma = 1.6$.

We treat the case where there are no variants as a reference system. The conditions are the same as the case of the ER network. The time evolution of the number of individuals of the three types (S, I, and R) is shown in Figure S4. We performed simulations for 100 samples. We plot the time evolution of all 100 samples in Figure S4(a), whereas the average of 100 samples is plotted in Figure S4(b).

We next investigate the effects of variants. Suppose that 10 susceptible individuals are infected with the variant due to external factors at $t = 10$. The variant is assumed to be 3.0 times more infectious with $\beta' = 0.8$. The average infection period is chosen as $1/\gamma' = 5.0$, which leads to $R_0' = 4.8$. The situation at $t = 10$ is that about 620 people are infected, 100 people are recovered, and 9280 people are not infected. This is similar to the case of ER network shown in Figure S2. In Figure S5, the number of individuals infected with the variant (I') and those who recovered from the variant (R') are shown in the dashed line. The overall behavior of the BA network is similar to the case of the ER network.

a b

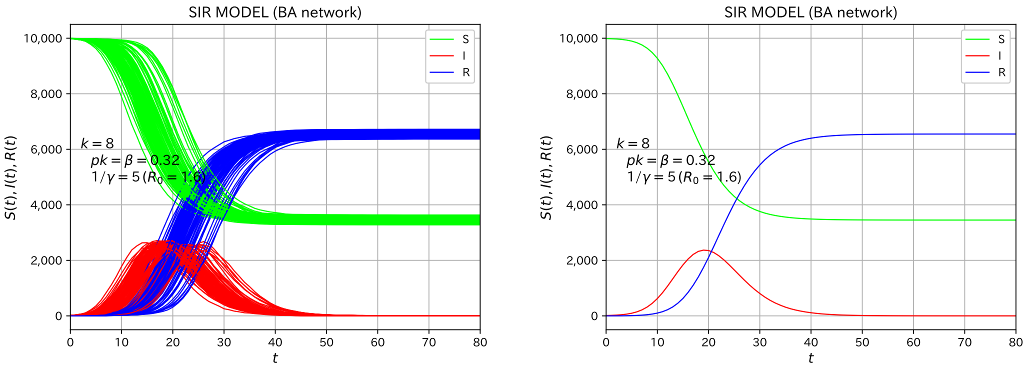

Figure S4. The simulational results of the microscopic SIR model on the BA network (reference system with no variants). (a) the plot of all 100 samples assumed to be infected with the virus with $R_0 = 1.6$. (b) the average over 100 samples. Initially, 10 individuals were set to be infected.

a b

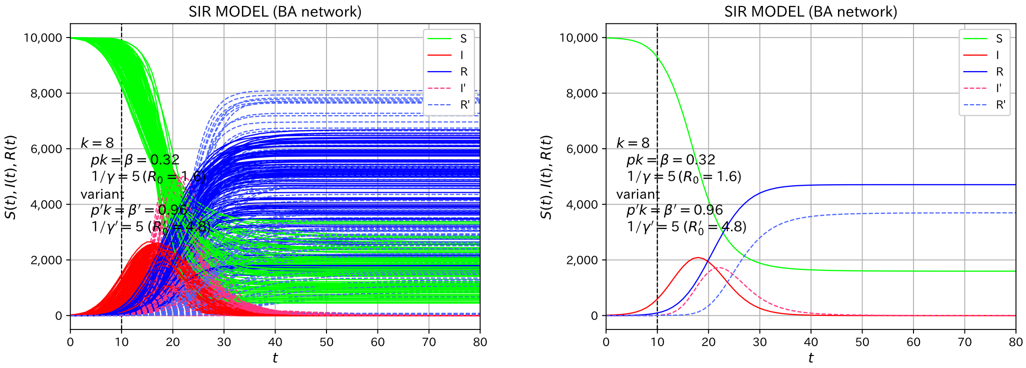

Figure S5. Effects of variants for the spread of the epidemic disease on the BA network. Suppose that 10 individuals are infected with the variant ($R_0' = 4.8$) by an external factor at $t = 10$. (a) the plot of all 100 samples. (b) the average of the 100 samples. The red and blue dashed lines indicate the values of variant.

Figure S6 shows the raw data of the spread of the epidemic disease with the variants with various strengths of infection. All the conditions are the same as in Figure S5 except for the value of β', that is R_0', of the variant. The values of R_0' are 4.4, 4.0, 3.6, 3.2, 2.8, 2.4, 2.0, and 1.6, which are 2.75, 2.5, 2.25, 2.0, 2.75, 1.5, 1.25, and 1.0 times that of the value of the original virus, respectively.

a1 b1

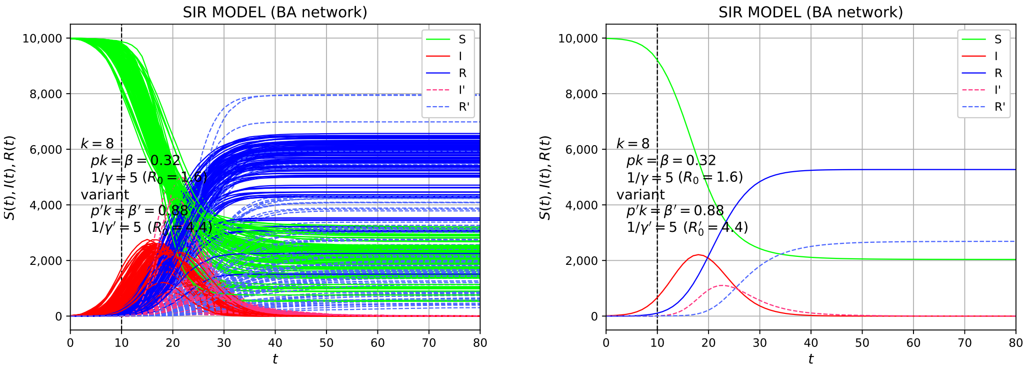

a2

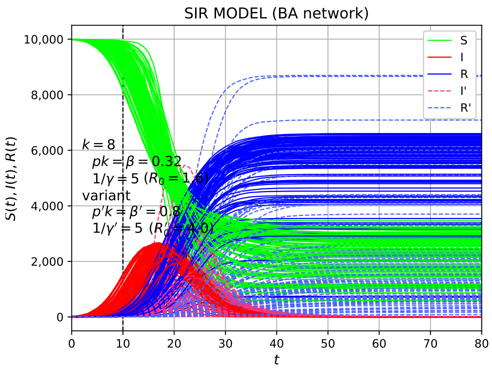

b2

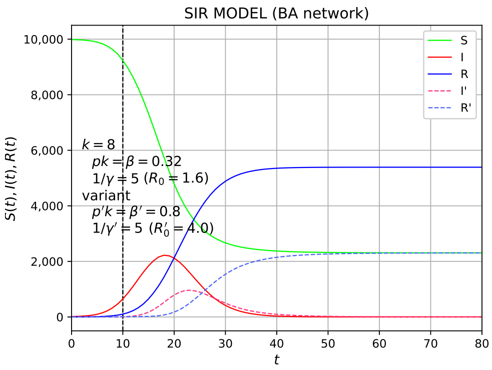

a3

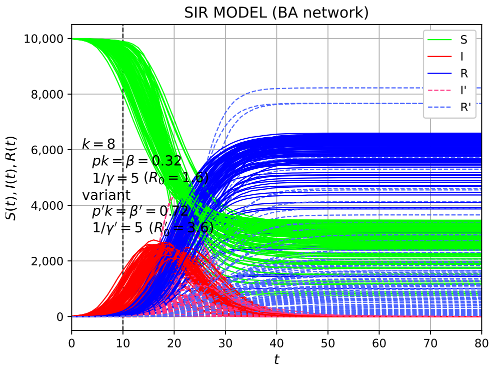

b3

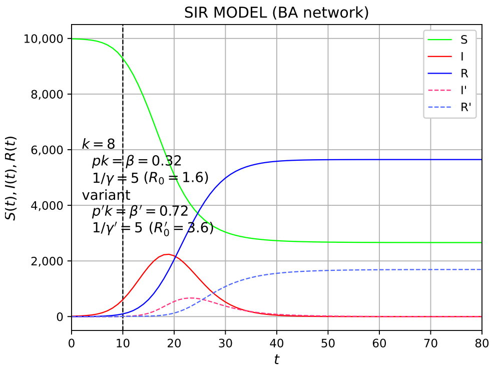

a4

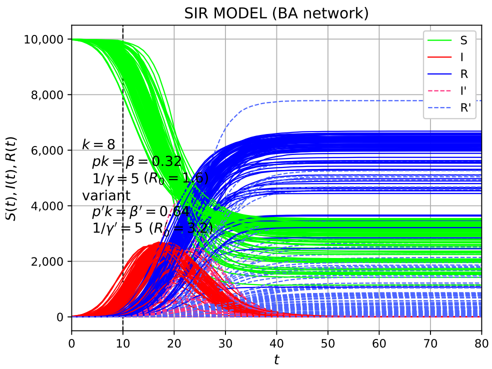

b4

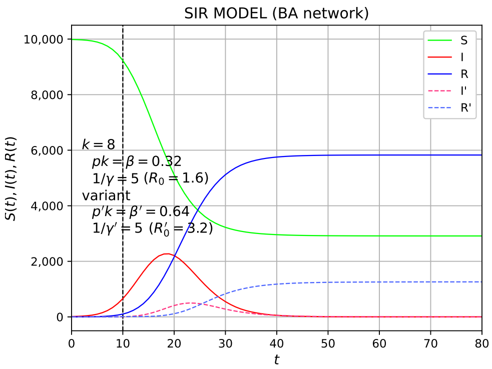

a5

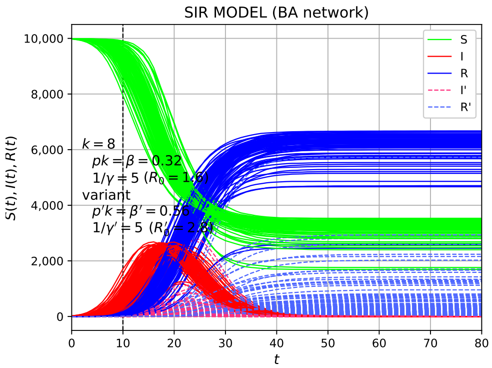

b5

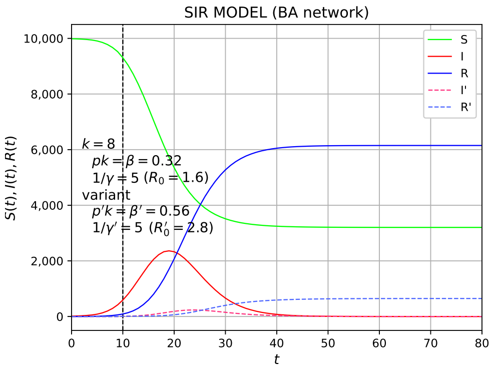

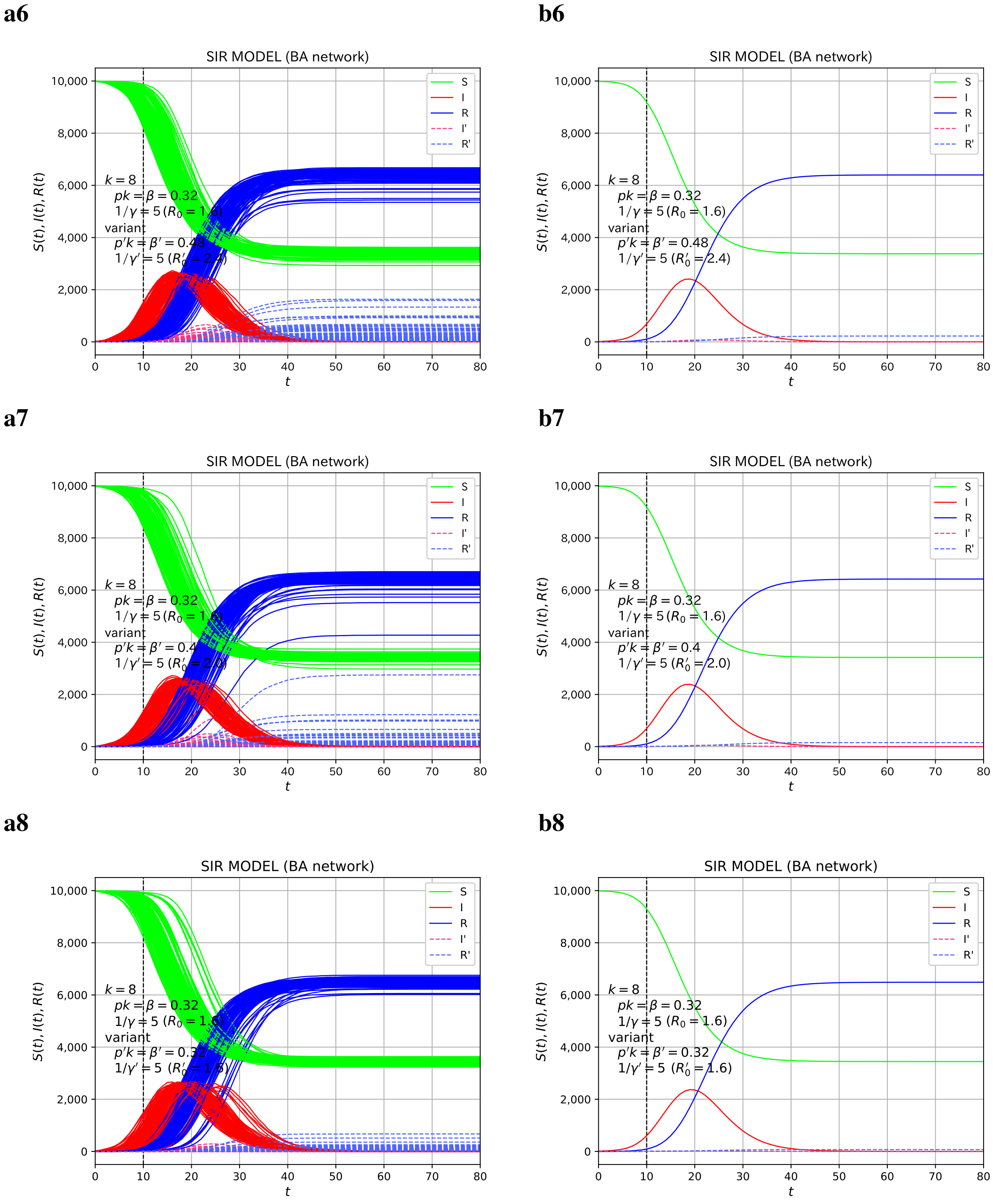

Figure S6. Effects of variants for the spread of the epidemic disease on the BA network. Suppose that 10 individuals are infected with the variant by an external factor at $t = 10$. (a1,b1) the plot of all 100 samples and the average of the 100 samples for $R_0' = 4.4$. (a2,b2) those for $R_0' = 4.0$. (a3,b3) those for $R_0' = 3.6$. (a4,b4) those for $R_0' = 3.2$. (a5,b5) those for $R_0' = 2.8$. (a6,b6) those for $R_0' = 2.4$. (a7,b7) those for $R_0' = 2.0$. (a8,b8) those for $R_0' = 1.6$. The red and blue dashed lines indicate the values of the variant.

References

1. Herrmann, H. A. & Schwartz, J.-M. Why COVID-19 models should incorporate the network of social interactions. *Phys. Biol.* **17**, 065008 (2020).
2. Okabe, Y. & Shudo, A. Microscopic Numerical Simulations of Epidemic Models on Networks. *Mathematics* **9**, 932 (2021).
3. Erdös, P. & Rényi, A. On Random Graphs I. *Publicationes Mathematicae* **6**, 290-297 (1959).

4. Erdös, P. & Rényi, A. On the evolution of random graphs. *Publ. Math. Inst. Hungar. Acad. Sci.* **5**, 17-61 (1960).

5. Barabási, A.-L. & Albert, R. Emergence of scaling in random networks. *Science* **286**, 509-512 (1999).

6. Marro, J. & Dickman, R. *Nonequilibrium Phase Transitions in Lattice Models (Collection Alea-Saclay: Monographs and Texts in Statistical Physics)*, (Cambridge, Cambridge University Press, 1999).

7. Mata, A. S. An overview of epidemic models with phase transitions to absorbing states running on top of complex networks. *Chaos* **31**, 012101 (2021).

8. Harris, T. E. Contact Interactions on a Lattice. *Ann. Probab.* **2**, 969-988 (1974).
